# Using machine-learning risk prediction models to triage the acuity of undifferentiated patients entering the emergency care system: a systematic review

**DOI:** 10.1186/s41512-020-00084-1

**Published:** 2020-10-02

**Authors:** Jamie Miles, Janette Turner, Richard Jacques, Julia Williams, Suzanne Mason

**Affiliations:** 1 Yorkshire Ambulance Service, Brindley Way, Wakefield, WF2 0XQ UK; 2 School of Health and Related Research, 3rd Floor, Regent Court (ScHARR), 30 Regent Street, Sheffield, S1 4DA UK; 3 University of Herfordshire, Hatfield, Herfordshire, UK

**Keywords:** Ambulance service, Emergency department, Machine learning, Triage, Patients

## Abstract

**Background:**

The primary objective of this review is to assess the accuracy of machine learning methods in their application of triaging the acuity of patients presenting in the Emergency Care System (ECS). The population are patients that have contacted the ambulance service or turned up at the Emergency Department. The index test is a machine-learning algorithm that aims to stratify the acuity of incoming patients at initial triage. This is in comparison to either an existing decision support tool, clinical opinion or in the absence of these, no comparator. The outcome of this review is the calibration, discrimination and classification statistics.

**Methods:**

Only derivation studies (with or without internal validation) were included. MEDLINE, CINAHL, PubMed and the grey literature were searched on the 14th December 2019. Risk of bias was assessed using the PROBAST tool and data was extracted using the CHARMS checklist. Discrimination (C-statistic) was a commonly reported model performance measure and therefore these statistics were represented as a range within each machine learning method. The majority of studies had poorly reported outcomes and thus a narrative synthesis of results was performed.

**Results:**

There was a total of 92 models (from 25 studies) included in the review. There were two main triage outcomes: hospitalisation (56 models), and critical care need (25 models). For hospitalisation, neural networks and tree-based methods both had a median C-statistic of 0.81 (IQR 0.80-0.84, 0.79-0.82). Logistic regression had a median C-statistic of 0.80 (0.74-0.83). For critical care need, neural networks had a median C-statistic of 0.89 (0.86-0.91), tree based 0.85 (0.84-0.88), and logistic regression 0.83 (0.79-0.84).

**Conclusions:**

Machine-learning methods appear accurate in triaging undifferentiated patients entering the Emergency Care System. There was no clear benefit of using one technique over another; however, models derived by logistic regression were more transparent in reporting model performance. Future studies should adhere to reporting guidelines and use these at the protocol design stage.

**Registration and funding:**

This systematic review is registered on the International prospective register of systematic reviews (PROSPERO) and can be accessed online at the following URL: https://www.crd.york.ac.uk/PROSPERO/display_record.php?ID=CRD42020168696

This study was funded by the NIHR as part of a Clinical Doctoral Research Fellowship.

## Introduction

### Rationale

Machine learning (ML) can be defined as ‘a set of methods that can automatically detect patterns in data, and then use uncovered patterns to predict future data, or to perform other kinds of decision making under uncertainty’ [1]. To date, ML has already proven effective at predicting outcomes for disease specific patients such as predicting bronchiolitis in infants and predicting whether trauma patients require a computerised tomography scan (CT) or have a cranio-cervical junction injury [2–4]. Other models have outperformed existing tools such as the Global Registry of Acute coronary Events (GRACE) and Thrombolysis In Myocardial Infarction (TIMI) risk tools at predicting cardiovascular risk [5, 6].

Initial triage at any stage of the Emergency Care System (ECS) has become challenging due to the increase in patients with varying levels of acuity [7]. Patients in a modern ECS have complex needs, which can often span mental health and social care [8].

Recently, there has been increased interest in combining ‘artificial intelligence’ with the Emergency Department for the purpose of initial triage [9–12]. However, this has been largely through the use of supervised learning algorithms, a sub-category of ML techniques [9]. The benefit of using these ML methods is they can identify non-linear relationships between candidate predictors and the outcome [11]. Furthermore, they can be embedded into electronic Patient Care Records (ePCR), removing the labour involved in triage and allowing for more complex models to be integrated [12].

The application of non-ML triage algorithms has previously led to the majority of patients being identified as mid-acuity. The Emergency Severity Index (ESI) is one such example [10, 11]. These triage systems can often have a clinical time-cost in their application [7]. In order for the benefits of triage algorithms to be actualised, the patient benefit at every acuity level has to be shortened. This means those with high acuity needs are treated quicker, those who are likely to be admitted are identified sooner and those with low acuity needs are discharged faster [10].

### Clinical role for the index test

The index test under investigation in this systematic review is any triage model that is applied by a clinician at the point of entry in the ECS. There are three possible entry points for patients. The first is when a patient calls the emergency medical service and is triaged by the Emergency Operations Centre (EOC). The second entry point is a face-to-face assessment by a paramedic on-scene. The third is on arrival to the Emergency Department (ED) [13]. A patient may enter at any of these points and also move through them all, being triaged multiple times. However the objective at each stage is the same: to stratify the acuity of an individual patient and allow the result to modify an ongoing care plan.

### Objectives

The primary objective of this review is to assess the accuracy of machine learning methods in their application of triaging the acuity of patients presenting in the Emergency Care System (ECS).

## Methods

This review followed the Preferred Reporting Items for Systematic Reviews and Meta-Analysis (PRISMA) statement. It is registered with PROSPERO (CRD42020168696).

### Eligibility criteria

#### Population

All patients presenting to the ECS who require a process of triage to discern the immediacy of care. The population cannot be differentiated by clinical severity or condition prior to the application of the triage tool. This is due to the index test under investigation being able to be applied to all incoming patients. The population can be differentiated by demographic variables such as age, as it is recognised, there is a difference in service need between younger and older populations [14–19].

#### Intervention (index test)

Machine learning algorithms that have been used to derive and internally validate a decision support tool. This includes commonly used methods such as logistic regression. However, for this review, the application of logistic regression must extend to making predictions in future data and not just uncovering patterns. The restriction to only derivation and internal validation studies is to ensure the method under investigation is clearly defined as opposed to an existing tool being externally validated in a subsequent population.

#### Comparison (reference test)

The reference test in this review is hierarchical. Preferably, there would be a decision support tool already used in the clinical setting identified in the paper as a comparator. In the absence of such, the study would include a clinician judgement. However, studies that have no comparator would also be accepted because derivation studies can often lack performance comparison with existing practice.

#### Outcome

For clarification, outcome has been divided into two parts. Prediction outcome and accuracy outcome.

### Prediction outcome

To be included in this review, the outcome has to be a triage acuity outcome for emergency care. Each included study is aiming to make a prediction about how ill a patient is, or how urgent their care need is. Because the methods of how these predictions are developed is under investigation in this systematic review, the prediction outcome was allowed to be broadened in order to capture all relevant studies. This may be strictly a triage level (such as the Emergency Severity Index 5–level) or a surrogate outcome such as predicting the need for critical care or hospitalisation.

### Model performance

For all the prediction models that have been included, their performance is described in terms of accuracy metrics reported in the final model performance. This includes discrimination (C-statistic), calibration (calibration plot, calibration slope, Hosmer-Lemeshow) and classification statistics (sensitivity, specificity, accuracy, positive predictive value (PPV), negative predictive value (NPV), likelihood ratio +/−). Some studies have used synonyms such as ‘precision’ instead of PPV, or ‘recall’ instead of sensitivity. For clarity in this review, all terms have been aligned to classification statistics identified in Steyerberg et al. [20].

### Information sources

On the 14th December 2019, the Medical Literature Analysis and Retrieval System Online (MEDLINE), the Cumulative Index to Nursing and Allied Health Literature (CINAHL), PubMed and the grey literature were searched. This included Google scholar and the IEEE arXiv.

### Search

A search strategy was developed through iteration and piloting. It was adapted from key words identified in the research questions and can be found in the supplementary material. The search strategy was used for MEDLINE, CINAHL and PubMed. This can be found in the supplementary material.

The search strategy was for the last 10 years only. This is due to clinical contexts and computer capabilities being rapidly changing industries and thus older studies have a higher risk of being void or outdated. The search also encompassed only those studies presented in the English language. This is due to limited access to interpretation services. Any non-English language studies were excluded at the selection stage.

### Study selection

Title screening was performed directly on source sites by JM and then exported to Endnote (version X9 for Windows) for abstract screening. This was subsequently fully second screened by JT. Then full text screening was performed by JM, with JT independently reviewing a random sample of 30% of the chosen included texts. Results were then compared with any disagreements being resolved by a third reviewer (SM). The data was selected from the studies retrieved during the searches using a visual schema transposed from the inclusion and exclusion criteria. This can be found in the supplementary material. There were four stages of selection based on the screening results of the studies. The first involved a population assessment, ensuring the study was set in the emergency care system and the patients are not differentiated clinically. The second stage involved intervention screening and ensuring the candidate variables were measured at triage (entry point). The third stage involved method screening, which in turn was subdivided into two sections, the first ensuring that machine learning was used to derive the model, and the second, ensuring that the methodological outcome was accuracy in prediction. The final stage involved outcome screening, ensuring that each selected study was setting out to risk-stratify patients. There was co-author validation of the included articles.

### Data collection process

Data was extracted using the Critical Appraisal and Data Extraction for Systematic Reviews of Prediction Modelling Studies (CHARMS) checklist [21]. This was completed in Microsoft Excel (2016) by JM. The total spreadsheet was reviewed by RJ. Any disagreements were mediated by a third reviewer (JT). Data extracted for each included study is provided in the supplementary material, as well as details regarding study quality assessment.

### Risk of bias and applicability

Risk of bias and applicability was undertaken using the Prediction model Risk of Bias Assessment Tool (PROBAST) [22]. A template was accessed at http://www.probast.org/wp-content/uploads/2020/02/PROBAST_20190515.pdf

It was completed for each model by JM and then checked by RJ. Any disagreements were mediated by a third reviewer (JT).

### Diagnostic accuracy measures

The principle diagnostic accuracy measures will be broadly covering three key areas. These are calibration, discrimination and classification of the final model within each study.

### Synthesis of results

The included studies were too heterogeneous to undertake a robust meta-analysis; therefore, a narrative synthesis was performed. This centred on discrimination as the most reported summary statistic of model performance. Where derivation and internal validation results have been presented separately in a model, only the internally validated performance is included in this review and not the apparent performance.

The included models were sub-grouped by outcome, and further by method. Median and IQR was used to illustrate the spread of the C-statistics within each method. The analysis plan was informed by the Cochrane Handbook for Systematic Reviews of Diagnostic Test Accuracy [23].

## Results

### Study selection

**Fig. 1:**
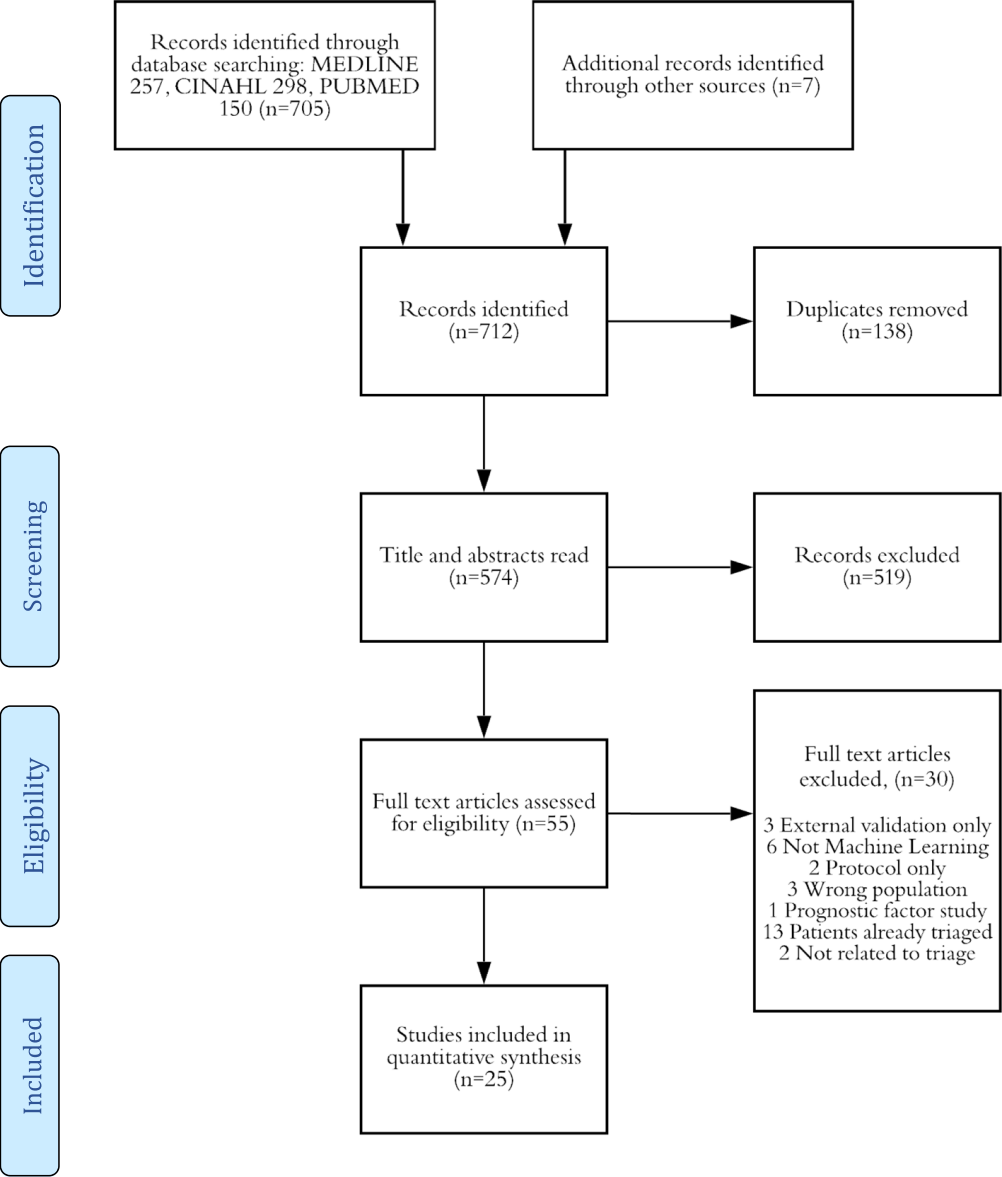
Study selection adapted from PRISMA [24].

All databases were searched on the 14th December 2019. There was a total of 712 studies identified from the database searching. This included 257 from MEDLINE, 298 from CINAHL and 150 from PubMed. Seven other sources from the grey literature were found. After title and abstract screening, 55 studies were taken through to eligibility screening. Thirty articles were excluded for the following reasons: 3 were external validation only, 6 were not machine learning, 2 were protocol only, 3 were studying the wrong population, 1 was a prognostic factor study, 13 had patients that were already triaged and 2 studies were not related to triage. This left a total of 25 studies included in this review. A PRISMA schematic diagram can be found below, and the PRISMA checklist can be found in the supplementary material [24]. Many studies investigated more than one machine learning technique, which meant that contained within the included studies was a total of 92 models to examine in this review (Fig. 1).

### Study characteristics

The three most common methods were logistic regression (36 models), tree-based methods (23) and neural networks (20). Other models included support vector machines (6), Bayesian models (5), a K-nearest neighbour model and a unique artificial neuro-fuzzy inference system. Of the 92 models, there were only 13 that were set in the pre-hospital setting. The rest were set in the ED at the point of triage. The two main outcomes that were being predicted by the studies were admission to hospital (53 models) or critical care outcome (28 models). Less common outcomes that appeared in these studies were predicting existing triage structures (9 models), and the prediction of whether a patient would be discharged from ED (3 models). Table 1 below summarises the key features of the included studies.

**Table 1:** Study characteristics

Author	Year	Country	Population	Outcome	Methods used	Predictors	Sample size	EPV	Method of testing
Azeez et al. [25]	2014	Malaysia	ED	Triage level	NN, ANFIS	20	2223		Random split sample (70:30)
Caicedo-Torres et al. [26]	2016	Spain	ED	Discharge	LR, SVM, NN	147	1205		Random split sample (80:20), 10-fCV
Cameron et al. [27]	2015	Scotland	ED	Hospitalisation	LR	9	215231		Random split sample (66:33), bootstrapping (10,000)
Dinh et al. [28]	2016	Australia	ED	Hospitalisation	LR	10	860832	9470	Random split sample (50:50)
Dugas et al. [29]	2016	USA	ED	Critical illness	LR	9	97000000	525	Random split sample (90:10), 10f-CV
Golmohammadi [30]	2016	USA	ED	Hospitalisation	LR, NN	8	7266	460.25	Split sample (70:30)
Goto et al. [31]	2019	USA	ED	Critical illness, hospitalisation	LR, LASSO, RF, GBDT, DNN	5	52037	32.60	Random split sample (70:30)
Hong et al. [32]	2018	USA	ED	Hospitalisation	LR, GBDT, DNN	972	560486	171.44	Random split sample (90:10)
Kim, D et al. [33]	2018	Korea	Prehospital	Critical illness	LR, RF, DNN	5	460865	3583.60	10f-CV
Kim, S et al. [34]	2014	Australia	ED	Hospitalisation	LR	8	100123	1074.86	Apparent performance
Kwon et al. (1) [35]	2018	Korea	ED	Critical illness, hospitalisation	DNN, RF	7	10967518	133667.89	Split sample (50:50), + external validation dataset
Kwon et al. (2) [36]	2019	Korea	ED	Critical illness, hospitalisation	DNN, RF, LR	8	2937078	14047.57	Split sample (50:50)
Levin et al. [37]	2018	USA	ED	Critical illness, hospitalisation	RF	6	172726	56.74	Random split sample (66:33), bootstrapping
Li et al. [38]	2009	USA	Pre-hospital	Hospitalisation	LR, NB, DT, SVM	6	2784		10f-CV
Meisel et al. [39]	2008	USA	Pre-hospital	Hospitalisation	LR	9	401		Bootstrap resampling (1000)
Newgard et al. [40]	2013	USA	Prehospital	Critical illness	CART	40	89261		Cross-validation
Olivia et al. [41]	2018	India	ED	Triage level	DT, SVM, NN, NB	8			10f-CV
Raita et al. [42]	2019	USA	ED	Critical illness, hospitalisation	LR, LASSO, RF, GBDT, DNN	6	135470	107	Random split sample (70:30)
Rendell et al. [43]	2019	Australia	ED	Hospitalisation	B, DT, LR, NN, NB, KNN	11	1721294	5521	10f-CV
Seymour et al. [44]	2010	USA	Prehospital	Critical illness	LR	12	144913	156	Random split sample (60:40)
van Rein et al. [45]	2019	Netherlands	Prehospital	Critical illness	LR	48	6859	3.4375	Separate external validation
Wang et al. [46]	2013	Taiwan	ED	Triage level	SVM	6	3000		10f-CV
Zhang et al. [47]	2017	USA	ED	Hospitalisation	LR, NN	25	47200	91.8	10f-CV
Zlotnik et al. [48]	2016	Spain	ED	Hospitalisation	NN	9	153970	614.5	10f-CV
Zmiri et al. [49]	2012	Israel	ED	Triage level	NB, C4.5	4	402		10f-CV

*ANFIS* Adaptive Neuro-Fuzzy Inference System, *B* Bayesian Network, *CART* Classification and Regression Tree, *DT* Decision Tree, *DNN* Deep Neural Network, *EPV* Events Per Variable, *GBDT* Gradient Boosted Decision Tree, *KNN* K-Nearest Neighbours, *LR* logistic regression, *LASSO* Least Absolute Shrinkage and Selection Operator, *NB* Naïve Bayes, *NN* Neural Network, *RF* Random Forest, *SVM* Support Vector Machine

There were 44 models derived in the USA, 18 in Korea, 14 in Australia, 5 in Spain, 4 in India, 2 in Malaysia, 2 in Israel and 1 in Taiwan, Scotland and the Netherlands. Eighty-four models were purely retrospective using existing registry or cohort data. Only 4 models included data collection that was prospective and there were 4 models that did not include whether the data source was retrospective or prospective. Sixty-three models were derived using data from multiple sites, whilst 25 models were developed using a single centre. Four models failed to publish this information.

### Risk of bias and applicability

**Fig. 2:**
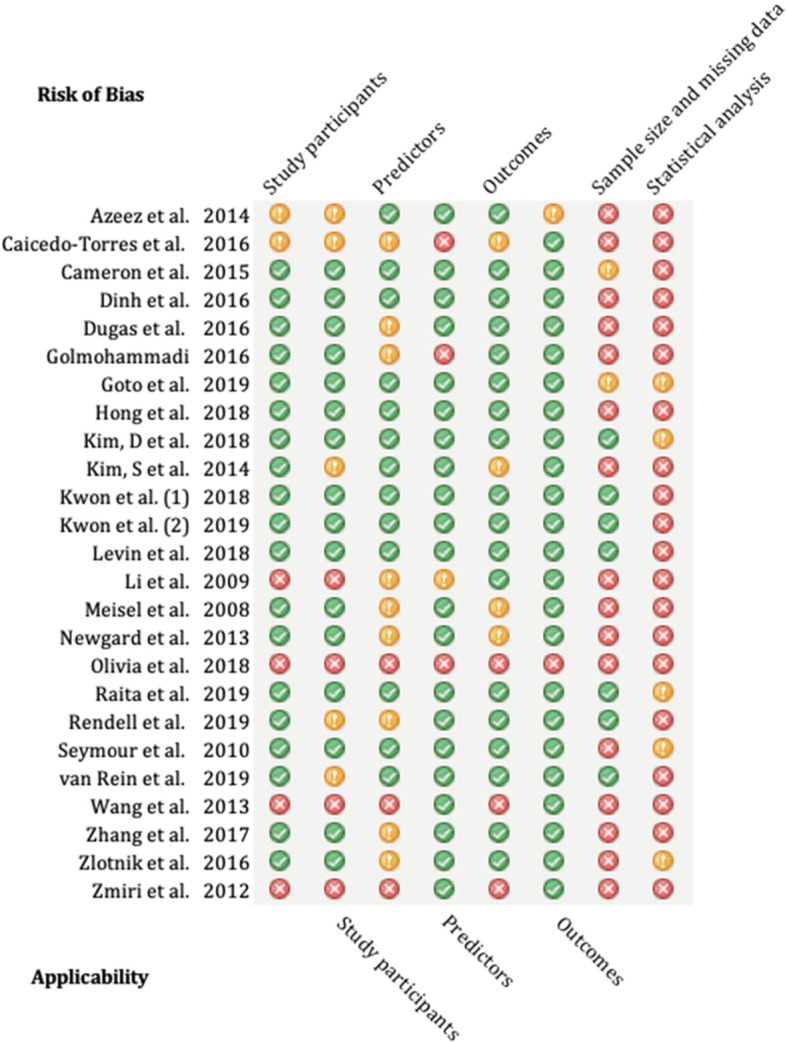
PROBAST assessment summary

There was a significant amount of incomplete reporting within the results. Only four models reported any calibration, mainly using the Hosmer-Lemeshow statistic [27, 44, 48]. One reported the *p* value of this, but not the statistic itself [27]. In terms of discrimination, there were 81 models that reported a concordance statistic (C -statistic), but of these, only 74 generated confidence intervals around this statistic. Only 47 models described classification statistics; however, these were incongruous between studies and only 1 study included the amount of true positive, true negative, false positive and false negative results. This makes it unfeasible to meta-analyse models which share the same population and outcome. A summary of the PROBAST assessment can be found in Fig. 2 and was adapted from Debray et al. [50]. When applying the PROBAST tool, there were only three studies which could be considered a low risk of bias [31, 33, 42]. This limits the benefit of grouping high vs low risk of bias studies. Most studies had low applicability concern, except for six studies [26, 30, 38, 41, 46, 49].

### Synthesis of results

#### Hospitalisation outcome

**Fig. 3:**
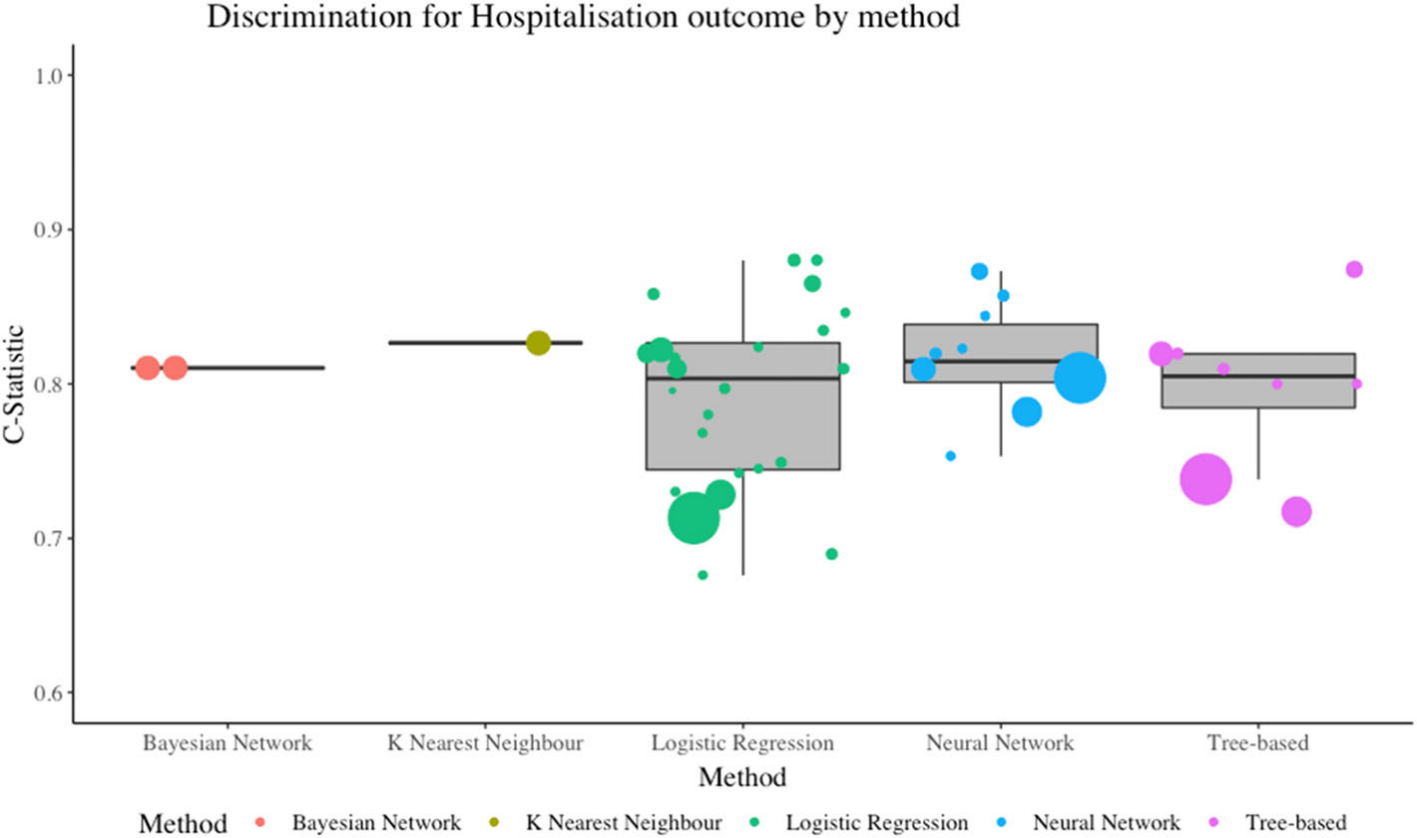
Discrimination for hospitalisation outcome by method

There was a total of 56 models which were predicting whether the patient was likely to be hospitalised as the outcome. Of these, 27 used logistic regression (two used the LASSO penalty term). Twelve studies used a neural network, 10 used a tree-based design, 3 Bayesian methods, 3 support vector machine models and one K-nearest neighbour. Only three models reported calibration in this outcome group [28, 48]. The most reported result was model discrimination using the C-statistic (also known as the area under the ROC curve, or AUC). Whilst the heterogeneity between models is too severe to undertake a meta-analysis, it was possible to cluster results by outcome and method. Figure 3 illustrates which machine learning methods were most able to differentiate between those with a positive outcome and those with a negative. The size of the data points is a normalised transformation of the sample size used to derive each model. Neural networks and tree-based methods both had a median C-statistic of 0.81 with their interquartile ranges (IQR) being 0.80-0.84 and 0.79-0.82 respectively. This compares to logistic regression which had a median C-statistic of 0.80 (IQR 0.74-0.83). The larger sample sizes generated smaller C-statistics. The three support vector machine models did not report the C-statistic. Classification was poorly reported with only 19 models publishing sensitivity and specificity, and only 10 of these also reporting confidence intervals. Twenty-one models reported accuracy, but only four of these had confidence intervals. Please refer to the CHARMS supplement for more details.

#### Critical illness

There were 28 models that used critical illness as an outcome measure. Eleven were logistic regression (one with LASSO penalty), 11 were tree-based and 6 were neural networks. There was an incongruency with the precise definition of critical illness, Table 2 highlights the differences within the definitions. Only one model in this group reported any calibration. They found that deep neural networks were the most discriminate with a C-statistic of 0.89 (95% CI 0.88-0.89). This compared to logistic regression and random forest modelling which both had the same result of 0.87 (95% CI 0.86-0.87).

**Table 2:** Critical care outcome definitions between studies

Study	Direct ICU	Death	Direct theatre	Direct pPCI	Severe sepsis	Mechanical intervention	ISS > 15	ISS > 16
Dugas et al.	**✓**	**✓**	**✓**	**✓**				
Goto et al.	**✓**	**✓**						
Kim D et al.		**✓**						
Kwon et al.	**✓**	**✓**						
Kwon et al. (2)	**✓**							
Levin et al.	**✓**	**✓**						
Newgard et al.								**✓**
Raita et al.	**✓**	**✓**						
Seymour et al.		**✓**			**✓**	**✓**		
van Rein et al.							**✓**	

*ICU* Intensive Care Unit, *pPCI* primary Percutaneous Coronary Intervention, *ISS* injury severity score

**Fig. 4:**
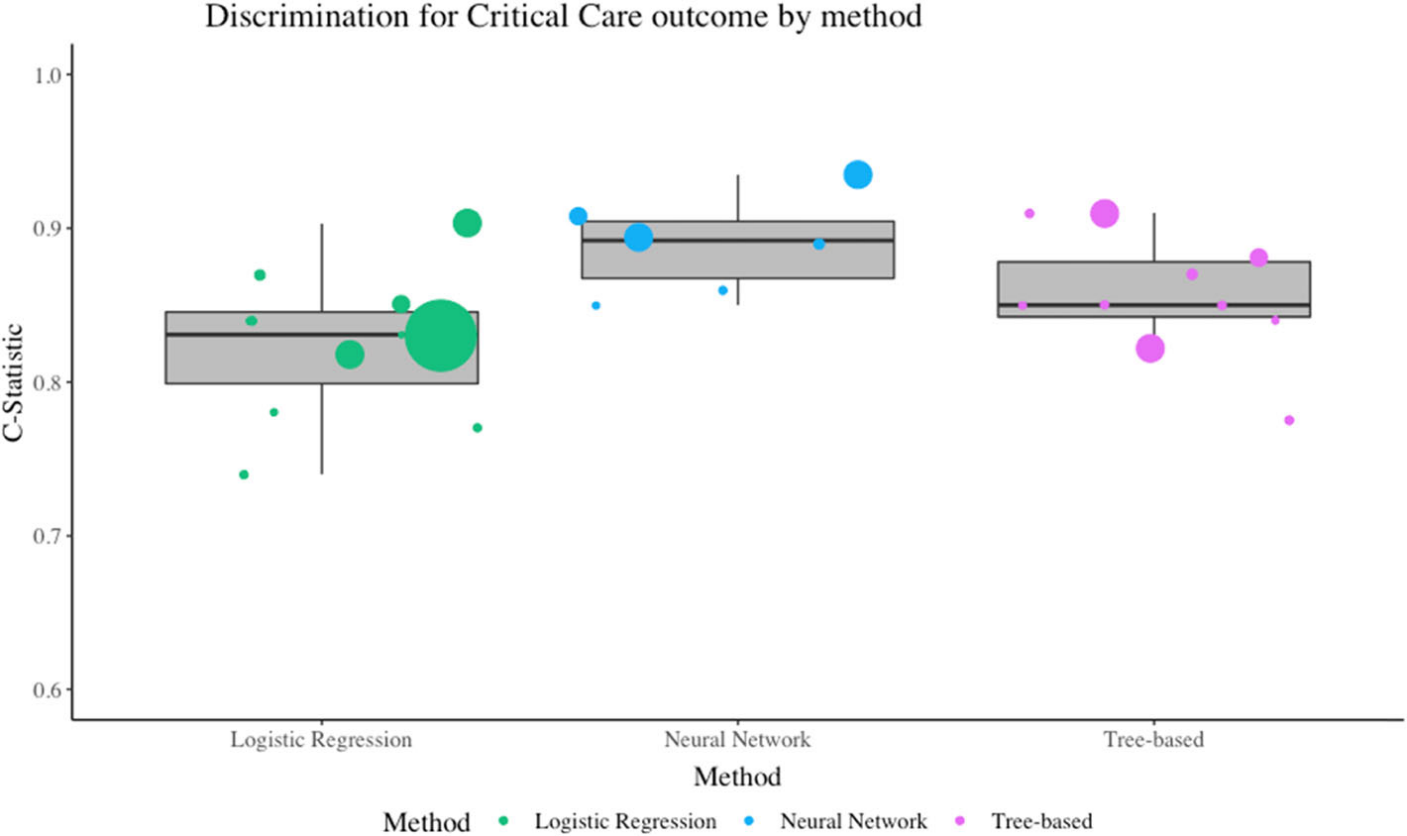
Discrimination for critical care outcome by method

The most common statistic was the C-statistic for discrimination. Figure 4 illustrates which methods were most discriminative at predicting a critical care outcome. As above, the sample size is represented by the size of the data point. Neural networks had a median of 0.89 (IQR 0.87-0.90) tree based had a median of 0.85 (IQR 0.84-0.88) and logistic regression had a median of 0.83 (IQR 0.80-0.85).

There were only 10 models from two studies that included classification metrics such as sensitivity and specificity with their associated confidence intervals [31, 42]. This makes comparison limited.

#### Discharge outcome

Three models from a single study used discharge related outcome measures [26]. The study focussed on predicting patients that would be discharged from the ED, and diverting them to a fast track service. They used logistic regression, support vector machines and neural networks for comparison. They did not report discrimination and only reported limited classification statistics [26]. They found that the neural network had the most precise estimates with a PPV (0.85) compared to the support vector machine and logistic regression (0.83 and 0.82). However, when examining the reported F1 score (PPV* sensitivity/PPV + sensitivity), logistic regression reported the most accurate estimate with an F1 score of 0.85, compared to the support vector machine (0.82) and the neural network (0.82).

#### Triage level outcome

Three studies that used machine learning to stratify patients into existing triage tools, all of which had a high risk of bias [25, 46, 49]. One focused on the Objective Primary Triage Scale (OPTS) in Malaysia [25]. This is a three tiered triage scale of emergent, urgent and non-urgent. They used neural networks and an artificial neuro-fuzzy inference system (ANFIS) to make predictions. There was no model calibration performed and the C-statistic did not have any confidence intervals. They did report accuracy and PPV for both methods and found the neural network had an accuracy of 0.84 (PPV 0.87) which was better performing than the ANFIS method (accuracy 0.6, PPV 0.61) [25]. Two studies used a local four level triage scale [46, 49]. One used Support Vector Machines with a Principle Component Analysis and a back propagated neural network, reporting an accuracy of 1.0 and 0.97 respectively [46]. The results in this study are likely to be biassed. The other study which examined a four-tiered triage scale used a naïve Bayes and a C4.5 tree-based classifier [49]. They only reported accuracy; however, they found that when they simplified the scale to be two grades, both models had higher prediction (average accuracy 71.37) than when it was four grades (52.94).

## Discussion

### Summary of included studies

In the last 10 years, there has been an increase in the number of prediction models that have utilised already existing methods in statistics and computer science. This may be due to the widespread availability of data worldwide. This systematic review identified 25 studies which aimed to derive a risk prediction model for triaging the acuity of undifferentiated patients in the emergency care system. The most common method was logistic regression with 36 models, but this was followed closely by both tree-based methods and neural networks. Most studies used hospital admission as an outcome for prediction. The objective of this review was to assess the accuracy of different machine learning methods. This was challenging due to differences in reporting how models were developed and evaluated. Furthermore, the reporting of the majority of models did not give enough information on model development, validation and performance which makes a critical appraisal difficult and a meta-analysis of accuracy stratified by the method almost impossible.

There have been common pitfalls amongst the included studies which will be discussed including the reference standard, the handling of candidate variables, and the analysis of performance.

### The reference standard

In evaluating the performance of a diagnostic model, it is important to compare the index test (the new model) with a ‘gold standard’, known as the reference standard. In practice, this could be subjective such as a clinician making a decision or deciding a triage level. Alternatively, it could be an objective standard such as an ICD-10 classification of disease, mortality or a clearly defined event [51].

Most studies that are determining the cross-sectional acuity of any given patient in emergency care have subjective reference standards. To illustrate, the Emergency Severity Index (ESI) 5-level triage is almost exclusively subjective and depends on the clinician undertaking the triage. A limitation of using this as a reference standard is inter-rater reliability can widely vary. A meta-analysis has shown that the inter-rater reliability of the ESI had an unweighted kappa of 0.786 (95% CI 0.745-0.821) [52]. Using subjective reference standards could lead to inherent problems maintaining the accuracy when transporting the model.

In contrast, Liu et al. undertook a study predicting cardiac arrest within 72 h of ED attendance [53]. A cardiac arrest is an empirical outcome measure and can be defined as “the abrupt loss of heart function in a person who may or may not have been diagnosed with heart disease” [54]. Liu prospectively collected data on 1386 participants and recorded whether or not they had a cardiac arrest within 72 h. In this example, the reference standard is a clearly defined outcome, which is not open for interpretation or subjectivity, and thus would provide a reliable benchmark to compare a derived model.

### Handling of candidate variables

Prior to developing a diagnostic model, it is important to consider which variables in the data are candidates for the final model. These candidate variables can be identified not only through subject knowledge or literature searching but also through statistical methods of examining the distribution or weighting [20]. A common problem with the included studies was how they reported the identification of candidate variables. Fifteen of the included studies provided a clear rationale, with data available at triage being the most common reason. Two studies used all the variables in the dataset, and eight studies provided no rationale at all.

It is also important to rationalise why there is a need to transform continuous variables given that it can be statistically inappropriate when developing prognostic models and leads to a significant loss of information [55]. Only 6 studies kept variables in their original format, whilst the remaining studies either categorised the variables (such as age) or did not describe the variables in a level of detail that an assessment could be made. Furthermore, no study elaborated on the linearity of the continuous variables and reported how they would model non-linear relationships (such as using fractional polynomials or restricted cubic splines) [56].

One of the benefits of using machine learning is the ability of performing feature selection during analysis [1]. The methods of undertaking feature selection can vary according to method, but the principle is beneficial to creating a simple model that can be embedded into practice. Methods such as deep neural networks can allow for fitting complex non-linear relationships through their architecture. The more hidden layers, the more complex the relationships. Univariable screening is not recommended as it does not account for any important collinearities between other candidate variables [57]. Despite this, it was used in 5 of the included studies.

### Reporting

The concordance statistic (C-statistic) was the most commonly reported and appeared in 81 out of 97 models. The C-statistic evaluates how discriminative a model is. For example, if a pair of subjects were selected at random (one with the outcome and one without), how often would the model classify both subjects correctly [58]. There were no significant differences in discrimination between methods, and all reported a range of C-statistics that performed well (above 0.7). However, reporting how discriminative a model is does not provide a full picture and the performance of the model should account for calibration. This is an assessment of accuracy or more specifically, how well the predictions matched the observed outcomes in the data [56]. If studies only report discrimination, then it does not help troubleshoot poor performance in a transported model. This is when the model is adopted in a new setting, such as a new hospital, or new country. Only five models reported any calibration, and two of these used the Hosmer-Lemeshow statistic [44, 48]. This is prone to poor interpretability and can be sensitive to sample size and grouping [56]. With machine learning methods, miscalibration can be adjusted when transporting a model to a different setting. Further ways to present accuracy are classification statistics. These include accuracy, sensitivity, specificity, positive predictive value (PPV), negative predictive value (NPV), and likelihood ratios.

No studies reported classification statistics in full. If they had published the true positive, false positive, true negative and false negative results for their model performance, a meta-analysis could have been performed [23].

Nearly all the studies had the potential of a high risk of bias due to the results being incomplete. More information is needed in order to make a robust judgement. The PROBAST statement recommends transparency in reporting and the transparent reporting of a multivariable prediction model for individual prognosis or diagnosis (TRIPOD) gives clear guidance on how to achieve this. Even though machine learning can be perceived as ‘black box’, this axiom is not entirely true. For example, DNN can obtain a matrix of parameter values and this can then be subsequently transformed into the ranking of variable importance. The reporting of model performance can still be generated [59, 60].

### Limitations in this review

This review identified and appraised all available literature; however, it did not directly contact authors for original data or further statistics. As such, the level of missing data in reporting which prevented the generation of a summary statistic remained throughout. This also had an impact on the risk of bias assessment. The Excerpta Medica database (EMBASE) was not used in this review as it was deemed too similar to MEDLINE.

## Conclusion

This systematic review has found that machine learning methods such as neural networks, tree-based, and logistic regression designs appear equal at triaging undifferentiated patients. However, the inconsistency and absence of information has significant implications on the risk of bias in all studies. Therefore no definitive answer can be drawn about the most accurate method. Future studies need to conform to reporting guidelines to ensure transparency and integrity of the models.

## Supplementary information

**Additional file 1:.**

## Data Availability

The datasets used and/or analysed during the current study are available from the corresponding author on reasonable request.

## Abbreviations

ANFIS: Adaptive Neuro-Fuzzy Inference System
AUC: Area under curve
B: Bayesian network
CART: Classification and regression trees
CHARMS: Critical Appraisal and Data Extraction for Systematic Reviews of Prediction Modelling Studies
CINAHL: The Cumulative Index to Nursing and Allied Health Literature
CT: Computerised tomography
DNN: Deep neural network
DT: Decision tree
ECS: Emergency Care System
ED: Emergency Department
EOC: Emergency Operations Centre
EPCR: Electronic patient care record
EPV: Events per variable
ESI: Emergency severity index
GBDT: Gradient boosted decision tree
GRACE: Global Registry of Acute Coronary Event
ICD-10: International Classification of Disease-10
IQR: Inter-quartile range
KNN: K-nearest neighbours
LR: Logistic regression
MEDLINE: Medical Literature Analysis and Retrieval System
ML: Machine learning
NIHR: National Institute for Health Research
NN: Neural network
NPV: Negative predictive value
OPTS: Objective Primary Triage Scale
PPV: Positive predictive value
PROBAST: Prediction model Risk Of Bias Assessment Tool
PROSPERO: The International Prospective Register of Systematic Reviews
RF: Random Forest
ROC: Receiver operating characteristic
SVM: Support vector machine
TIMI: Thrombolysis in Myocardial Infarction
TRIPOD: Transparent Reporting of a multivariable prediction model for Individual Prognosis Or Diagnosis
