# Supplementary material for: Using machine-learning risk prediction models to triage the acuity of undifferentiated patients entering the emergency care system: a systematic review
Source: Diagn Progn Res. 2020 Oct 2;4:16. doi: 10.1186/s41512-020-00084-1 (PMC7531169; doi:10.1186/s41512-020-00084-1)
Supplement: Supplementary file 1 — Additional file 1:. [file 41512_2020_84_MOESM1_ESM.docx]

## Search term identification

| **Key terms** | **Alternative terms** | **Subject headings** |
| --- | --- | --- |
| Machine Learning | “Machine Learn*”  ML  “Artificial Intelligence”  AI  Deriv*  Valid* | Supervised Machine Learning/  Unsupervised Machine Learning/  Machine Learning/  Algorithms/  Logistic Models/ |
| Clinical triage | “Clinic* Triag*”  Triag*  “Clinic* Classif*”  Classif*  “Clinic* sort*”  Sort” | Clinical Triage/ |
| Patient acuity | “Patient severity”  Prognos*  Predict*  Rule* | Patient Acuity/  Ingui filter*  Haynes broad filter** |
| Urgent and Emergency care | “*Emergenc* care*”  Emergenc*  “Urgent Care”  Urgent  Prehospital  Pre-hospital  Pre hospital  “Emergency Department”  ED  “Accident and Emergency”  “Accident & Emergency”  A&E  Ambulanc*  “Ambulanc* Serv*  EMS  “Emergency Medical Service” | Emergency Medical Services/  Emergency Medicine/  Emergency Treatment/  Emergencies/  Ambulatory care/  Ambulances/  Emergency Medical Tags/  Emergency Medical Technicians/  Emergency Responders/  Emergency Service, Hospital/ |
| *Ingui filer | (Validat* OR Predict*.ti. OR Rule*) OR (Predict* AND (Outcome* OR Risk* OR Model*)) OR ((History OR Variable* OR Criteria OR Scor* OR Characteristic* OR Finding* OR Factor*) AND (Predict* OR Model* OR Decision* OR Identif* OR Prognos*)) OR (Decision* AND (Model* OR Clinical* OR Logistic Models/)) OR (Prognostic AND (History OR Variable* OR Criteria OR Scor* OR Characteristic* OR Finding* OR Factor* OR Model*)) | |
| *Haynes broad filter | (Predict*[tiab] OR Predictive value of tests[mh] OR Scor*[tiab] OR Observ*[tiab] OR Observer  variation[mh]) | |

## Search strategy

Last 10 years – Clinical contexts and computer capabilities are both rapidly changing industries and thus older studies have a higher risk of being void or outdated. Can review this.

English only – Don’t have access to interpretation services and therefore including foreign language studies could lead to misinterpretation.

**MEDLINE via Ovid 257 results**

(Machine Learn* OR ML OR Artificial Intelligence OR AI OR Big data OR Gaussian process OR Cross-validation OR Cross validation OR Crossvalidation OR Regularized logistic OR Linear discriminant analysis OR LDA OR Random forest OR Na#ve Bayes* OR Least Absolute selection shrinkage operator OR elastic net OR LASSO OR RVM OR relevance vector machine OR pattern recognition OR Computational Intelligence OR Computational Intelligences OR Machine Intelligence OR Knowledge Representation OR Knowledge Representations OR support vector OR SVM OR pattern classification OR Supervised Machine Learning/ OR Unsupervised Machine Learning/ OR Machine Learning/ OR Algorithms/ OR Logistic Models/)

AND

(Clinic* Triag* OR Triag* OR Clinic* Classif* OR Classif* OR Clinic* sort* OR Sort* OR electro* triag* OR Digital triag* OR Clinical Triage/)

AND

(Patient severity OR Prognos* OR Predict* OR Rule* OR Patient Acuity/)

AND

(Emergenc* care* OR Urgent and Emergency Care OR Urgent & Emergency Care OR UEC OR Emergenc* OR Urgent Care OR Urgent OR Prehospital OR Pre-hospita OR Pre hospital OR Emergency Department OR ED OR Accident and Emergency OR Accident & Emergency OR A&E OR Ambulanc* OR Ambulanc* Serv* OR EMS OR Emergency Medical Service OR Emergency Medical Services/ OR Emergency Medicine/ OR Emergency Treatment/ OR Emergencies/ OR Ambulatory care/ OR Ambulances/ OR Emergency Medical Tags/ OR Emergency Medical Technicians/ OR Emergency Responders/ OR Emergency Service, Hospital/)

**CINAHL via EBSCO 298 results**

(Machine Learn* OR ML OR Artificial Intelligence OR AI OR Big data OR Gaussian process OR Cross-validation OR Cross validation OR Crossvalidation OR Regularized logistic OR Linear discriminant analysis OR LDA OR Random forest OR Na#ve Bayes* OR Least Absolute selection shrinkage operator OR elastic net OR LASSO OR RVM OR relevance vector machine OR pattern recognition OR Computational Intelligence OR Computational Intelligences OR Machine Intelligence OR Knowledge Representation OR Knowledge Representations OR support vector OR SVM OR pattern classification OR Supervised Machine Learning/ OR Unsupervised Machine Learning/ OR Machine Learning/ OR Algorithms/ OR Logistic Models/)

AND

(Clinic* Triag* OR Triag* OR Clinic* Classif* OR Classif* OR Clinic* sort* OR Sort* OR electro* triag* OR Digital triag* OR Clinical Triage/)

AND

(Patient severity OR Prognos* OR Predict* OR Rule* OR Patient Acuity/)

AND

(Emergenc* care* OR Urgent and Emergency Care OR Urgent & Emergency Care OR UEC OR Emergenc* OR Urgent Care OR Urgent OR Prehospital OR Pre-hospita OR Pre hospital OR Emergency Department OR “ED” OR Accident and Emergency OR Accident & Emergency OR “A&E” OR Ambulanc* OR Ambulanc* Serv* OR “EMS” OR Emergency Medical Service OR Emergency Medical Services/ OR Emergency Medicine/ OR Emergency Treatment/ OR Emergencies/ OR Ambulatory care/ OR Ambulances/ OR Emergency Medical Tags/ OR Emergency Medical Technicians/ OR Emergency Responders/ OR Emergency Service, Hospital/)

**PubMed 150 results**

(Machine Learn* OR ML OR Artificial Intelligence OR AI OR Big data OR Gaussian process OR Cross-validation OR Cross validation OR Crossvalidation OR Regularized logistic OR Linear discriminant analysis OR LDA OR Random forest OR Na#ve Bayes* OR Least Absolute selection shrinkage operator OR elastic net OR LASSO OR RVM OR relevance vector machine OR pattern recognition OR Computational Intelligence OR Computational Intelligences OR Machine Intelligence OR Knowledge Representation OR Knowledge Representations OR support vector OR SVM OR pattern classification OR Supervised Machine Learning[mh] OR Unsupervised Machine Learning[mh] OR Machine Learning[mh] OR Algorithms[mh] OR Logistic Models[mh])

AND

(Clinic* Triag* OR Triag* OR Clinic* Classif* OR Classif* OR Clinic* sort* OR Sort* OR electro* triag* OR Digital triag* OR Clinical Triage[mh])

AND

(Patient severity OR Prognos* OR Predict* OR Rule* OR Patient Acuity[mh])

AND

(Emergenc* care* OR Urgent and Emergency Care OR Urgent & Emergency Care OR UEC OR Emergenc* OR Urgent Care OR Urgent OR Prehospital OR Pre-hospita OR Pre hospital OR Emergency Department OR “ED” OR Accident and Emergency OR Accident & Emergency OR “A&E” OR Ambulanc* OR Ambulanc* Serv* OR “EMS” OR Emergency Medical Service OR Emergency Medical Services[mh] OR Emergency Medicine[mh] OR Emergency Treatment[mh] OR Emergencies[mh] OR Ambulatory care[mh] OR Ambulances[mh] OR Emergency Medical Tags[mh] OR Emergency Medical Technicians[mh] OR Emergency Responders[mh] OR Emergency Service, Hospital[mh])

**782 results after duplicates removed.**

## Visual schematic of inclusion criteria for systematic review


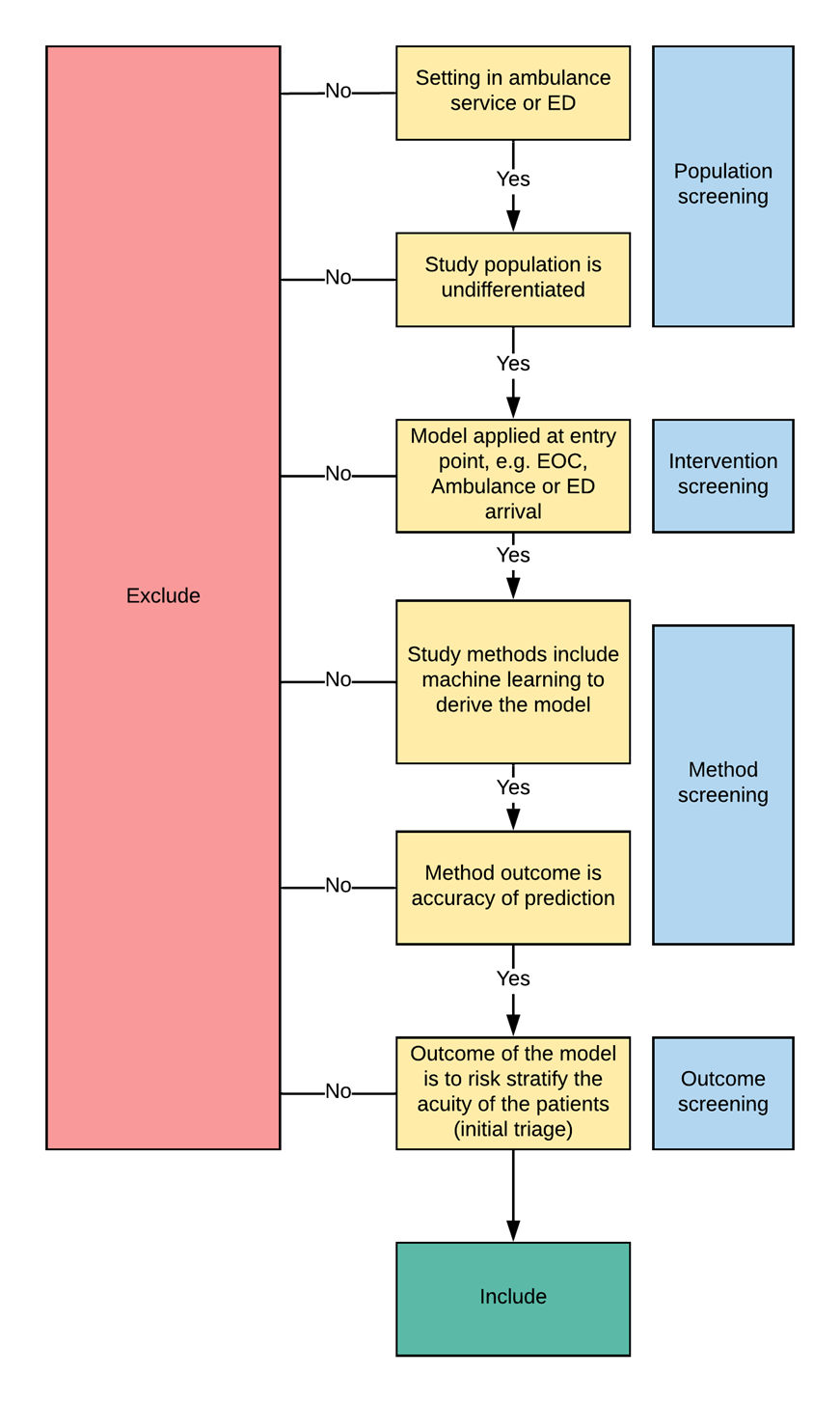


## Study selection adapted from PRISMA^60^


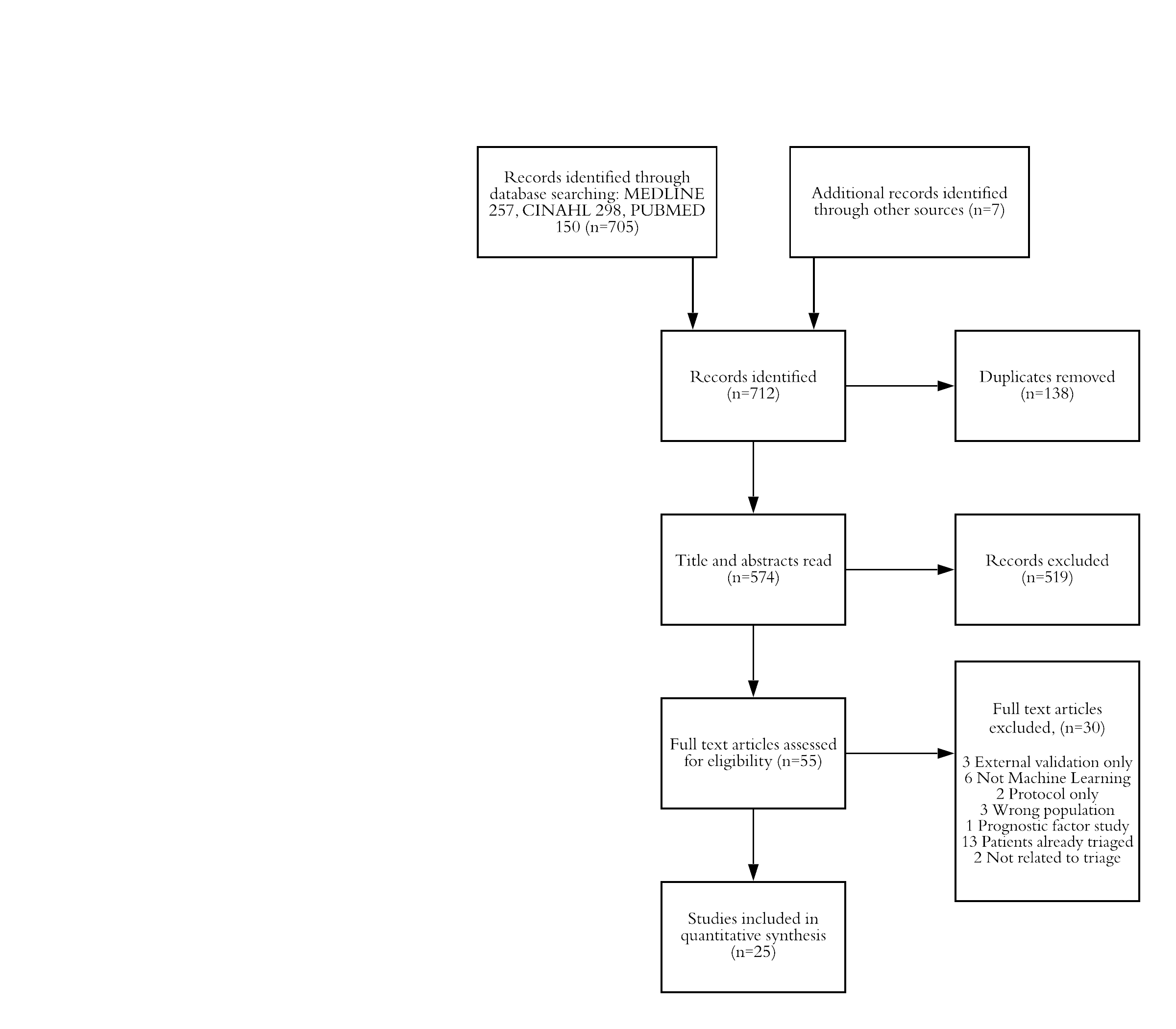


## Identification

## Screening

## Eligibility

## Included
